# Aspiration Pneumonitis Caused by Polyethylene Glycol-Electrolyte Solution Treated with Conservative Management

**DOI:** 10.1155/2014/872634

**Published:** 2014-05-18

**Authors:** Ricardo A. Mosquera, Mark McDonald, Cheryl Samuels

**Affiliations:** High Risk Children Comprehensive Care Clinic, University of Texas Health Science of Houston, 6410 Fannin Street, Suite 500, Houston, TX 77030, USA

## Abstract

Polyethylene glycol (PEG) electrolyte solution, Golytely, is an osmotic laxative commonly used in preoperative bowel cleansing. In this case report, a 9-year-old boy developed aspiration pneumonitis following accidental infusion of PEG solution into his right lung following migration of his nasogastric tube (NGT). Hypoxemia and tachypnea without respiratory failure were observed after infusion. Because PEG is a nonabsorbable toxic material, previous case reports have advocated for the performance of bronchoalveolar lavage (BAL) in the treatment of PEG pneumonitis. With close monitoring, our patient was able to be successfully treated without the need for invasive interventions including BAL or intubation. Generalizations about PEG absorption in the lung based on its permeability in the gastrointestinal tract should not deter the use of more conservative treatment in the appropriate patient.

## 1. Introduction

Polyethylene glycol (PEG) is a complex organic polymer that can be combined with sodium sulfate to form a highly efficacious osmotic laxative with a variety of medical applications [1]. This PEG-electrolyte solution is commonly used in preoperative bowel cleansing as it has been demonstrated that PEG is both effective and well tolerated in pediatric and adult populations [1, 2]. A major disadvantage of using PEG in bowel preparation is the unpleasant taste and the relatively large volume that must be ingested [1, 3]. This limitation can be overcome by administering the solution via nasogastric tube [1]. Common adverse effects of PEG include nausea, vomiting, and bloating though more serious gastrointestinal (GI) complications like pancreatitis can occur in addition to anaphylaxis, angioedema, and ventricular arrhythmia [4]. Additionally, the introduction of PEG solution into the lungs causes significant inflammation and an intraluminary fluid shift resulting in pulmonary edema [3]. These complications can be life-threatening and even fatal [3, 5–10]. Current treatment recommendations from previous case reports include the use of prophylactic antibiotics, IV corticosteroids, and early bronchoscopy with bronchoalveolar lavage (BAL) and intubation with ventilation [6, 8, 9, 11]. In this report, we describe a pediatric patient who developed aspiration pneumonitis secondary to accidental PEG aspiration and was successfully treated with careful monitoring, IV antibiotics, and IV steroids without BAL. His symptoms disappeared after four days. Chest radiograph revealed complete resolution of right lower lobe opacities 7 days later.

## 2. Case Presentation

A 9-year-old Hispanic boy with spina bifida, hydrocephalus status post-VP shunt with normal neurological cognitive function, and neurogenic bladder with ileovesicostomy was scheduled for bladder augmentation and revision of his urinary diversion. He was admitted to the hospital for preoperative bowel preparation consisting of polyethylene lavage (1400cc over 4 hours) via nasogastric feeding tube (NGT) with a kangaroo feeding pump. The NGT placement was confirmed with auscultation. Shortly after the infusion was begun, the patient developed significant coughing and gagging with small amount of emesis of clear fluid. The infusion was immediately stopped and a radiograph showed placement of the NG tube in the right lung. A review of the feeding pump revealed the patient had received a total of 283 mL of solution. The primary care provider and the pediatric and pulmonary services were notified and consulted.

**Figure 1:**
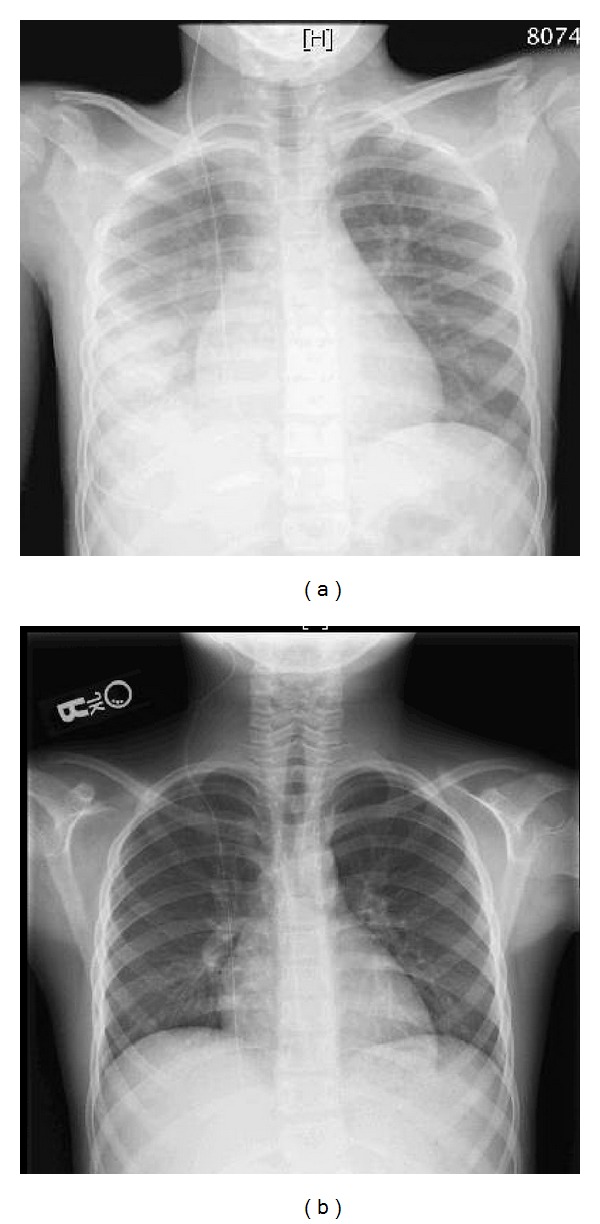
(a) CXR obtained immediately after nasogastric infusion of 283 mL of PEG in the right lung revealed right lower lobe opacities. (b) CXR 7 days after infusion showed significant clearance of right lower lobe opacities.

Prior to the incident the patient's vitals were stable with a heart rate of 80 beats/minute, blood pressure 90/64 mm Hg, respiratory rate 18 breaths/minute, and oxygen saturation 100% in room air. When the infusion was stopped, the patient's vitals were mildly elevated with a heart rate of 126 beats/min, blood pressure 111/55 mm Hg, a respiratory rate 28 breaths/min, and oxygen saturation 93% in room air. On exam the patient had decreased breath sounds over the right lung field with no crackles or wheezing and did not appear to be in significant distress. A chest X-ray showed a right lower lobe aspiration pneumonitis (Figure 1). IV clindamycin was begun and the patient was transferred to the Pediatric IMU (intermediate medical unit) for continuous cardiopulmonary monitoring with recommendations to start supplemental oxygen and intravenous steroids if his condition deteriorated. Overnight, the patient had an increase in tachypnea and his oxygen desaturated to 87–89% on room air. He subsequently received 2 liters of oxygen via nasal cannula to maintain oxygen saturations above 92% and IV Solu-Medrol was begun at a dose of 2 mg/kg/day. A subsequent X-ray was performed the following day that showed worsening of his pneumonitis though his PCO2 and pH remained within normal limits and he appeared clinically stable. Two days after the ingestion, the patient was transitioned to room air and his condition began to steadily improve. The patient was discharged 4 days after the infusion and a final chest X-ray obtained demonstrated complete resolution of pneumonitis (Figure 1).

## 3. Discussion

In the present case, the patient had respiratory compromise secondary to the accidental infusion. However, his respiratory compromise did not deteriorate to respiratory failure. With careful, continuous cardiopulmonary monitoring, the decision was made not to perform bronchoscopy with BAL. This decision was made with the family after all options were discussed in detail. They expressed concern for him to be taken to the bronchoscopy procedure room and to be sedated/anesthetized. Authors in previous case studies have recommended BAL as the treatment of choice for aspiration pneumonitis from PEG infusion [6, 11]. One assumption that supports this recommendation is that PEG is nonabsorbable [6]. However, this may be a generalization based on the permeability of PEG in the gastrointestinal tract which significantly differs from the permeability in the pulmonary epithelium [2]. Given that in the current case the aspiration pneumonitis resolved without invasive intervention to remove the offending agent, we speculate, based on this experience, that aspirated PEG can be absorbed or neutralized. This interpretation is consistent with the finding that in the rodent lung only half of a solution of 3.4 kDa PEG (similar size to PEG) remained in the alveoli after 9-10 hours [12, 13]. While bronchoscopy is a relatively safe procedure with a risk of approximately 5% for minor complications and <2% for major complications, it is still an invasive procedure that requires sedation and may not be necessary in an otherwise clinically stable patient [14]. Our finding is supported by another case study in which an adult patient was only given antibiotics, corticosteroids, and diuretics following PEG aspiration and the clinical and radiographic manifestations of his pneumonitis resolved after 3 days [4]. In most of the case reports the use of BAL and/or intubation with ventilation for treatment of PEG aspiration are recommended. These interventions may have been very appropriate as the conditions of the patients in these cases were more severe [3, 7–11]. Given the absence of designed studies assessing the costs and benefits of using bronchoscopy with BAL or intubation with ventilation for treatment of PEG aspiration, we recommend that the stability of the patient be an important determinant when deciding on treatment modalities. Additionally, we believe that treatment decisions should not be rooted in generalizations about the absorption of PEG based solely on its permeability in the GI tract.
